# Risk factors and economic impact of long-term nursing care after major trauma

**DOI:** 10.3389/fpubh.2025.1535784

**Published:** 2025-03-18

**Authors:** Ling-Wei Kuo, Po-Chuan Ko, Chien-An Liao, Yu-Tung Huang, Chi-Tung Cheng, Yu-Hsin Wang, Chun-Hsiang Ouyang, Jen-Fu Huang

**Affiliations:** ^1^Department of Trauma and Emergency Surgery, Chang Gung Memorial Hospital, Linkou Medical Center, Taoyuan, Taiwan; ^2^Center for Big Data Analytics and Statistics, Chang Gung Memorial Hospital, Linkou Medical Center, Taoyuan, Taiwan; ^3^Institute of Biomedical Engineering, College of Medicine and College of Engineering, National Taiwan University, Taipei, Taiwan; ^4^National Center for Geriatrics and Welfare Research, National Health Research Institutes, Yunlin, Taiwan; ^5^Division of Trauma and Emergency Surgery, Department of General Surgery, Jen-Ai Hospital, Taichung, Taiwan

**Keywords:** health care economics, long-term care, medical expenses, national health insurance, major trauma

## Abstract

**Introduction:**

The public could bear a heavy economic burden for trauma survivors needing long-term nursing care, especially in countries such as Taiwan that have universal health insurance coverage. The purpose of this study was to analyze the data from the National Health Insurance Research Database and to assess reimbursement to trauma patients with long-term sequelae who need nursing care.

**Methods:**

This study included all patients who suffered major trauma (injury severity score ≥ 16) in Taiwan from 2003 to 2007. Ten years of follow-up were analyzed. Patients aged 18 to 70 who survived for more than 1 year after the index admission were enrolled. Patients who needed long-term nursing care (LTC) were compared with those who did not (non-LTC). Basic demographics and short-term outcomes were analyzed, and the 10-year healthcare expenditure was calculated.

**Results:**

The study included 10,642 patients, 1,718 in the LTC group and 8,924 in the non-LTC group. Age, comorbidities, spinal cord injury, longer mechanical ventilation, longer ICU length of stay (LOS), and longer hospital LOS were identified as independent risk factors for LTC. The median 10-year healthcare expenditure was 43,979 USD in the LTC group vs. 9,057 USD in the non-LTC group (*p* < 0.001).

**Conclusions:**

16.14% of major trauma patients needed LTC at least 1 year after being discharged. The resource they receive in Taiwan is prominently less than the same patient group in the US. The NHI should invest more in post-discharge care for major trauma patients to optimize their care.

## Introduction

Trauma has long been a worrisome issue in the healthcare system in Taiwan. In this country, with a population of 23 million, roughly 6,000 people suffer from major trauma [injury severity score (ISS) more than 16] each year, and this could even be an underestimation. The in-hospital mortality of these patients in Taiwan is ~14%, indicating that most of them need medical attention afterward (1). Many of the survivors of major trauma cannot reach the general health status of the average population, even years after the injury (2). Aside from physical impairment, the high expenditure puts a burden on the healthcare system.

The financial burden for the survivors after the index admission could be high. The overall 6-month post-discharge medical encounters for trauma patients in the United States cost ~20,000 US dollars (UDS) (3), about 50% of which is funded by private health insurance (4). However, Taiwan is a country well known for its universal health insurance for its inhabitants. The National Health Insurance (NHI) program was initiated in 1995, and the government runs it as a single-payer insurance system with mandatory enrollment. Currently, >99% of Taiwan's population (~23 million residents) receives medical care through the NHI (5). Therefore, the economic accountability of the public sector is substantial.

Long-term nursing care (LTC) requires special attention. LTC patients have higher mortality rates, so it is essential to invest in resources to improve the outcomes (6–8). However, evidence regarding the scale of the expenditure is scarce. To our best knowledge, the limited literature regarding this topic was focused on specific populations, such as veterans from wars (9) or traumatic brain injury patients (10). A comprehensive understanding of the LTC of the general trauma population is still largely unknown. To tailor a care plan to trauma patients with long-term nursing needs, we must first examine the current budget to understand the overall picture. The long-term, nationwide, and universal nature of Taiwan's NHI is a suitable context in which to investigate the country's healthcare expenditures. The NHI research database (NHIRD) comprises all claims pertaining to visits, procedures, and prescription medications and includes anonymous eligibility and enrollment information. Here, we analyzed the data from the NHIRD to identify the risk factors for LTC and its following economic impact on the NHI.

## Methods

### Data

Data regarding medical services are collected by the National Health Insurance Administration and entered into the NHIRD. In this study, complete medical and surgical records from 2003 to 2017 in the database were analyzed, including all claims from the emergency department (ED), in-hospital admissions, and outpatient clinics.

### Study cohort

This retrospective observational study included all patients with major trauma suffered in Taiwan from 2003 to 2007, and a 10-year follow-up was conducted on this cohort. The National Health Act stipulates that severely ill or injured insured should be exempt from copayment when they receive treatment for their disease or injury. Hence, the catastrophic illness certificate (CIC) is issued for these severe patients to decrease the financial burden from medical treatment, including outpatient clinic visits, ED visits, and hospital admissions. Major trauma with ISS ≥16 has be an eligibility criterion for the CIC, and therefore, we used the trauma CIC as a proxy for major trauma patients. Note that the NHIRD does not contain the original ISS data, so the application for trauma CIC must be done by medical facilities. The NHI then performs strict chart reviews before issuing a CIC to prevent unnecessary compensation and extra expenses. As a result, the CIC can serve as an accurate guide for identifying the appropriate patients for our purposes. We also have to clarify that major trauma patients have been eligible for the CIC application since the beginning of the NHI, but before 2003, there was no unique coding for such patients, so there was no way to identify them from the NHIRD. As a result, we chose 2003 as the beginning of our study period.

After locating patients with major trauma, we ruled out patients who had previously received CIC for other conditions: malignancy, bleeding disorders, autoimmune diseases, end-stage renal disease (ESRD), severe psychological disorders, congenital metabolic disorders, congenital anomalies, major burn, transplantation recipients, major neurologic or motor neuron disorders, long-term mechanical ventilation, short bowel syndrome, myasthenia gravis, immunodeficiencies, severe liver cirrhosis, leprosy, the toxic effect of arsenic, and other rare diseases. These conditions could heavily interact with trauma and its long-term outcome, so they were excluded from the analysis.

Next, we eliminated patients who did not survive for more than 1 year after the index trauma incident. From our previous study regarding the long-term survival of major trauma in Taiwan, almost 90% of the 10-year mortality occurs within 1 year after discharge (11). Therefore, we chose 1 year as our threshold. Lastly, we excluded patients who were < 18 years of age. Patients older than 70 years of age were also excluded because, during the study period, the life expectancy in Taiwan was ~80 years (12). If patients older than 70 were included, the result of this 10-year follow-up might be skewed by natural deaths. Finally, patients between the ages of 18 and 70 years with no severe disease who suffered a major trauma from 2003 to 2007 and survived more than 1 year after were included.

### Variables and outcome

This study aimed to identify the risk factors for long-term nursing care (LTC) and the subsequent economic impact on the NHI. LTC was defined as being admitted to a chronic ward or having skilled nursing care in the outpatient fashion 1 year after discharge from the index admission. The functional outcomes at post-injury year one is a common milestone used in trauma-related studies (13, 14), and studies have shown that the functional improvement after 1 year is marginal (15–17). Therefore, we chose needing LTC after 1 year as our cut point. The record of chronic ward admission could be found in the Inpatient Expenditures by Admissions file in the NHIRD, and the record of skilled nursing care could be found in the Ambulatory Care Expenditures by Visits file. Preexisting factors, including age, sex, and underlying comorbidities, were analyzed, and comorbidities were quantified by the Charlson Comorbidity Index (CCI). The analysis also included specific injuries categorized into seven groups by body region: head, spinal cord, thoracic, abdominal and retroperitoneum, pelvis, extremities, and thermal injuries. These injuries were sorted by the International Classification of Diseases, Ninth Revision (ICD-9) codes, which are listed in the Supplemental File. We also considered short-term outcomes and complications during the index admission. Complications, including acute coronary syndrome (ACS), stroke, hospital-acquired pneumonia (HAP), urinary tract infection (UTI), wound complications, need for tracheostomy, need for dialysis, and need for cardiopulmonary cerebral resuscitation (CPCR), were identified in the admission dataset of the NHIRD. Short-term outcomes, including prolonged mechanical ventilation (PMV), prolonged intensive care unit (ICU) length of stay (LOS), and prolonged total LOS, were also evaluated. PMV was defined as mechanical ventilation for more than 21 consecutive days (18); prolonged ICU LOS (PICULOS) was defined as an ICU stay ≥14 days (19), and prolonged total LOS (PLOS) was admission for > 30 days, a quality index commonly used in the NHI (20). Post-discharge long-term survival was also calculated since it was a critical reference for the analysis of health expenditure.

### Data analysis

The LTC and non-LTC groups were compared to find any difference in the preexisting physical and socioeconomic factors, specific injury types, and short-term outcomes and complications. The 10-year healthcare expenditure per person was compared between the two groups to assess the scale of the economic impact of their nursing care. In the subgroup analysis for health expenditure, patients were further divided by the destination of LTC. These destinations include non-LTC, home-LTC, facility-LTC, and mixed LTC. The definition of home-LTC is that patients receive LTC from home-based medical care, whereas the definition of facility-LTC is patients receiving LTC in long-term facilities. Mixed LTC implies that the patients switched between home care and facilities. Healthcare expenditure only included the cost of medical services reimbursed by the NHI, including inpatient, outpatient, and emergency services. The expenditure cost was estimated by the NHI Global Budget Point Value Database. Each service, procedure, and medication in every medical facility has a corresponding point value, and 1 point should be approximately equal to 1 New Taiwan Dollar (TWD). Therefore, the cost of personal health equipment, over-the-counter medication, or other out-of-pocket expenses was not included. TWD were exchanged to USD under the average exchange rate in the study period, in which 1 USD equaled 30 TWD. The continuous variables were processed with the Mann–Whitney U test or Kruskal-Wallis test, where appropriate. The categorical variables were processed with the χ^2^ test. Long-term survival was analyzed by the Kaplan-Meier method. Logistic regression was performed to determine the factors that predicted LTC after index admission and variables with *p* < 0.05 were selected for multivariate analysis. All statistical analyses were performed with SAS version 9.4 (SAS Institute Inc., Cary, North Carolina, USA). A 2-sided *p* < 0.05 was considered statistically significant.

### Ethics approval and consent to participate

This study adhered to the Declaration of Helsinki. It has been reviewed and approved by the Chang Gung Medical Foundation Institutional Review Board (IRB) under IRB No. 202000280B0C501.

## Results

**Figure 1:**
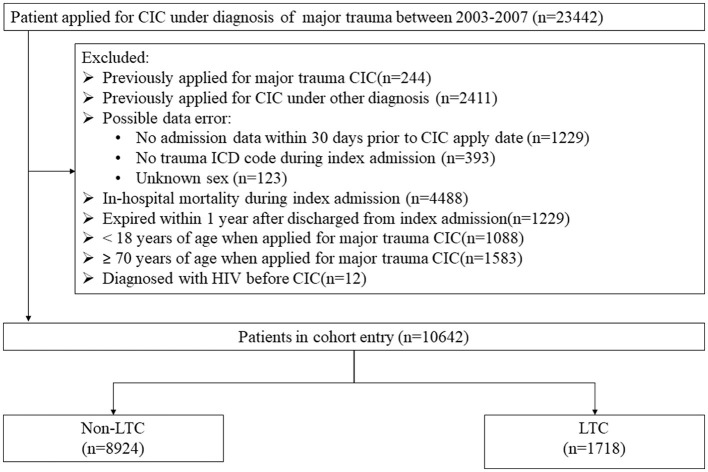
The algorithm of the data extraction from the NHIRD. From 2003 to 2007, 23,442 patients were initially identified from the NHIRD. After excluding patients with missing data and those who did not meet the inclusion criteria, 10,642 patients were included in the analysis. CIC, Catastrophic illness certificate; ICD, International Classification of Diseases; HIV, human immunodeficiency virus; LTC, Long-term nursing care.

At first, 23,442 patients were possibly eligible from the NHIRD. After applying the exclusion criteria, 10,642 patients were included in our statistical analysis (Figure 1). Table 1 shows the enrolled patients' basic demographics, injury types, short-term outcomes, and complications. The median age of these patients was 42 (28–55), and nearly 75% were male. The median CCI (excluding age) was 0. The median pre-injury income level was 640 USD in our cohort, so the participants were divided into low-income ( ≤ 640 USD per month) and high-income (>640 USD per month) groups. In our study cohort, 67.36% were in the low-income group, and 32.64% belonged to the high-income group. The most common injury site was the head (76.65%), followed by the extremities (24.26%) and chest (22.63%). The proportions of PMV, PICULOS, and PLOS were 12.98, 30.32, and 41.78%, respectively.

**Table 1:** Major trauma patients in 2003–2007 (*n* = 10,642).

**Variables**	
Age (median, IQR)	42 (28–55)
Sex (male, %)	7,970 (74.89%)
**CCI (without age)**	
0	7,861 (73.87%)
>0	2,781 (26.13%)
**Injury types**	
Head injuries (%)	8,157 (76.65%)
Thoracic injuries (%)	2,408 (22.63%)
Abdominal and retroperitoneal injuries (%)	1,462 (13.74%)
Pelvic injuries (%)	584 (5.49%)
Spinal cord injuries (%)	947 (8.9%)
Extremity injuries (%)	2,603 (24.46%)
Thermal injuries (%)	50 (0.47%)
**Index admission outcomes and complications**	
Tracheostomy (%)	26 (0.24%)
Dialysis (%)	53 (0.5%)
CPCR (%)	75 (0.7%)
Stroke (%)	111 (1.04%)
Acute coronary syndrome (%)	15 (0.14%)
**Prolonged mechanical ventilation (%)**	
No (< 21 days)	9,261 (87.02%)
Yes (≥21)	1,381 (12.98%)
**Prolonged ICU LOS (%)**	
No ( ≤ 14 days)	7,415 (69.68%)
Yes (>14 days)	3,227 (30.32%)
**Prolonged LOS (%)**	
No ( ≤ 30 days)	6,196 (58.22%)
Yes (>30 days)	4,446 (41.78%)
Post-discharge 10-year healthcare expenditure (mean, USD)	25,558 ± 40,650.7
Post-discharge 10-year healthcare expenditure (median, USD)	11,902 (4,448–30,768)

Continuous variables are presented in median/interquartile range (IQR); categorical variables are presented in numbers and percentage. Post-discharge 10-year health expenditure are presented in mean/standard deviation and median/IQR.

Among these patients, 1,718 needed LTC, leaving 8,924 in the non-LTC group (Table 2). Compared to the non-LTC group, patients in the LTC group were significantly older (median age: 49 vs. 41, *p* < 0.0001) and more often had comorbidities (33.88% CCI > 0 vs. 24.64%, *p* < 0.0001). The proportions of head injuries (78.75 vs. 76.24%, p = 0.0243) and spinal cord injuries (15.6 vs. 7.61%, *p* < 0.0001) were higher in the LTC group, but the proportions of chest injuries (17.75 vs. 23.57%, *p* < 0.0001), abdominal and retroperitoneal injuries (5.59 vs. 15.31%), pelvic injuries (3.49 vs. 5.87%), and extremity injuries (18.98 vs. 25.52%) were lower in the LTC group.

**Table 2:** Comparison of Non-LTC group to LTC group.

**Variables**	**Major trauma patients in 2003–2007 (*****n*** = **10,642)**		***p*-value**
	**Non-LTC (*****n*** = **8,924)**	**LTC (*****n*** = **1,718)**	
Age (median, IQR)	41 (27–54)	49 (36–60)	< 0.0001
Sex (male, %)	6,674 (74.79%)	1,296 (75.44%)	0.5697
CCI (without age)			< 0.0001
0	6,725 (75.36%)	1,136 (66.12%)	
>0	2,199 (24.64%)	582 (33.88%)	
**Injury types**			
Head injuries (%)	6,804 (76.24%)	1,353 (78.75%)	0.0243
Thoracic injuries (%)	2,103 (23.57%)	305 (17.75%)	< 0.0001
Abdominal and retroperitoneal injuries (%)	1,366 (15.31%)	96 (5.59%)	< 0.0001
Pelvic injuries (%)	524 (5.87%)	60 (3.49%)	< 0.0001
Spinal cord injuries (%)	679 (7.61%)	268 (15.6%)	< 0.0001
Extremity injuries (%)	2,277 (25.52%)	326 (18.98%)	< 0.0001
Thermal injuries (%)	39 (0.44%)	11 (0.64%)	0.0243
**Index admission outcomes and complications**			
Tracheostomy (%)	18 (0.2%)	8 (0.47%)	0.2593
Dialysis (%)	46 (0.52%)	7 (0.41%)	0.0572
CPCR (%)	58 (0.65%)	17 (0.99%)	0.5603
Stroke (%)	76 (0.85%)	35 (2.04%)	0.1234
Acute coronary syndrome (%)	9 (0.1%)	6 (0.35%)	< 0.0001
Prolonged mechanical ventilation (%)			< 0.0001
No (< 21 days)	7,995 (89.59%)	1,266 (73.69%)	
Yes (≥21)	929 (10.41%)	452 (26.31%)	
Prolonged ICU LOS (%)			< 0.0001
No ( ≤ 14 days)	6,589 (73.83%)	826 (48.08%)	
Yes (>14 days)	2,335 (26.17%)	892 (51.92%)	
Prolonged LOS (%)			< 0.0001
No ( ≤ 30 days)	5,568 (62.39%)	628 (36.55%)	
Yes (>30 days)	3,356 (37.61%)	1,090 (63.45%)	
Post-discharge 10-year healthcare expenditure (mean)	580,326 ± 1026680.5	1731585 ± 1618536.5	< 0.0001
Post-discharge 10-year healthcare expenditure (median)	271,713 (111354.5–671,124)	1,319,374 (746,868–2,195,265)	< 0.0001

**Figure 2:**
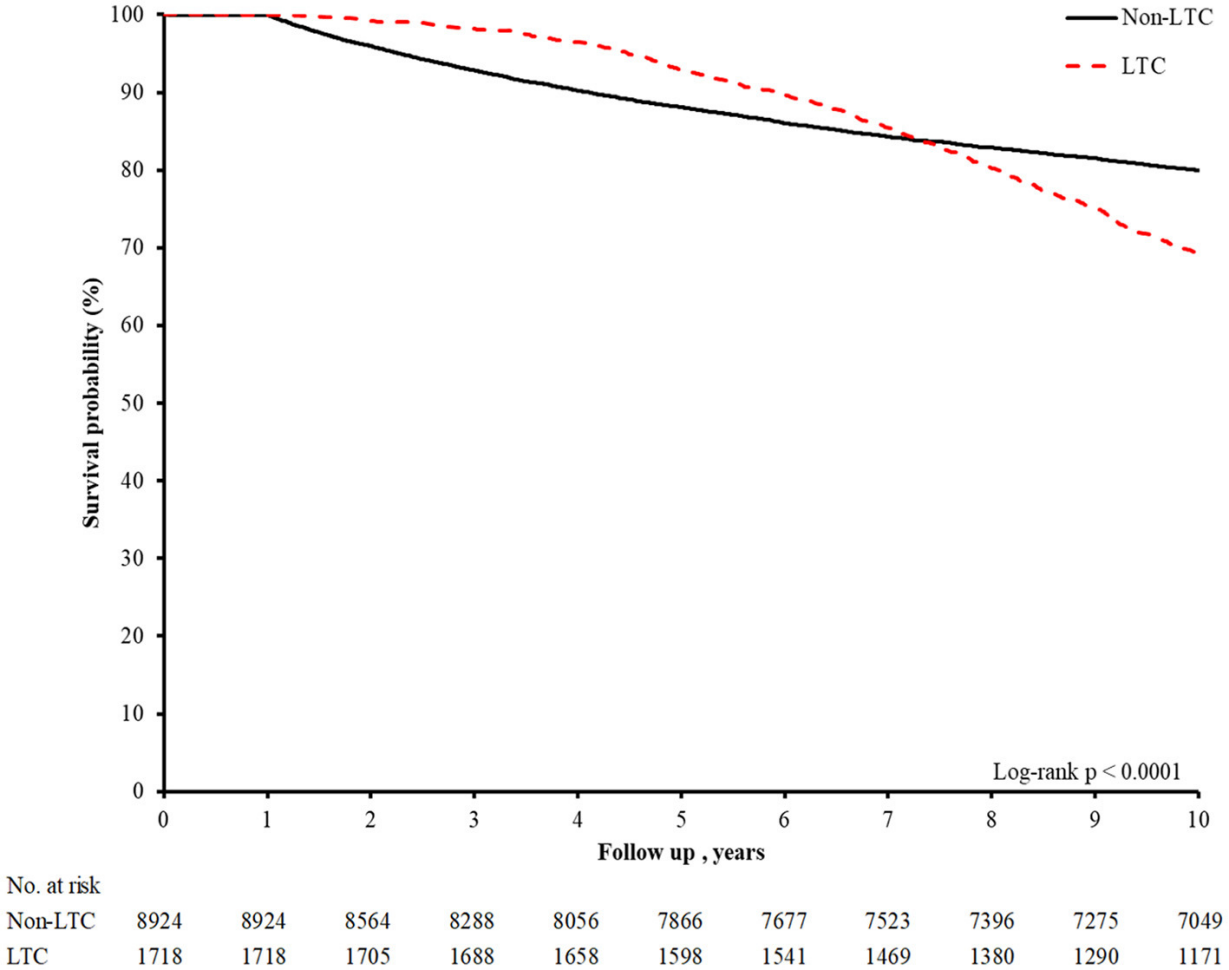
The 10-year survival rate for LTC and non-LTC group. LTC, Long-term nursing care.

Regarding complications and short-term outcomes, the LTC group had more patients suffering from stroke (2.04 vs. 0.85%, *p* < 0.0001), ACS (0.35 vs. 0.06%, p = 0.0236), HAP (23.52 vs. 11.47%, *p* < 0.0001), and UTI (13.74 vs. 6.11%, *p* < 0.0001). The proportion needing PMV (26.31 vs. 10.41%, *p* < 0.0001), the PICULOS (51.92 vs. 26.17%, *p* < 0.0001), and the PLOS (63.45 vs. 37.61%, *p* < 0.0001) were all higher in the LTC group. The median 10-year healthcare expenditure per person was 43,979 USD in the LTC group and 9,057 USD in the non-LTC group (*p* < 0.0001). We also calculated the mean 10-year healthcare expenditure per person, and the numbers were 57,720 USD for the LTC group and 19,344 USD for the non-LTC group. Long-term survival is presented in Figure 2. The 10-year survival rates for LTC and non-LTC groups were 68.16 and 78.99%, respectively, but the survival rate was superior for the LTC group before post-discharge year seven. In the subgroup analysis for different long-term destinations (Tables 3, 4), we have noticed that the median health expenditure increased by the order of non-LTC (9,057 USD), home-LTC (39,211 USD), facility-LTC (48,743 USD), and mixed LTC (66,388 USD).

**Table 3:** Subgroup analysis on post-discharge health expenditure for different destinations.

	**Post-discharge 10-year healthcare expenditure (median, USD)**	***P*-value**
Overall (*n* = 12,221, 100%)	13,558 (5,004–33,226)	< 0.001
Non-LTC (*n* = 10,099, 82.64%)	9,057 (3,712–22,371)	
LTC-home (*n* = 1,239, 10.14%)	39,211 (23,804–60,308)	
LTC-facility (*n* = 656, 5.37%)	48,743 (23,851–79,417)	
LTC-mixed (*n* = 227, 1.86%)	65,388 (38,437–102,668)	

**Table 4:** Analysis on post-discharge health expenditure for patients older than 70 years of age.

	**Post-discharge 10-year healthcare expenditure (median, USD)**	***P*-value**
Overall (*n* = 1,580, 100%)	24,117 (12,366–50,965)	< 0.001
Non-LTC (*n* = 1,176, 74.43%)	21,234 (10,416–43,352)	
LTC-home (*n* = 277, 17.53%)	34,557 (20,752–60,252)	
LTC-facility (*n* = 97, 6.14%)	33,635 (18,827–80,156)	
LTC-mixed (*n* = 227, 1.86%)	49,759 (31,611–109,409)	

LTC, Long-term nursing care; CCI, Charlson Comorbidity Index; ICU, intensive care unit; LOS, length of stay.

Many factors were associated with LTC in the univariate logistic regression model (Table 5), whereas in the multivariate model, only each year of age (OR = 1.018, 95% CI 1.014–1.022), the presence of comorbidities (CCI > 0; OR = 1.163, 95% CI 1.028–1.317), spinal cord injury (OR = 1.945, 95% CI 1.617–2.340), HAP (OR = 1.313, 95% CI 1.137–1.517), UTI (OR = 1.478, 95% CI 1.242–1.760), PMV (OR = 1.253, 95% CI 1.068–1.470), PICULOS (OR = 1.844, 95% CI 1.595–2.132), and PLOS (OR = 1.663, 95% CI 1.457–1.897) were identified as independent risk factors for LTC. Chest injury (OR = 0.833, 95% CI 0.719–0.965), abdominal and retroperitoneal injury (OR = 0.499, 95% CI 0.395–0.631), and extremity injury (OR = 0.836, 95% CI 0.726–0.962) remained negatively associated with the risk of LTC.

**Table 5:** Risk/protective factor analysis for LTC.

**Variables**	**Univariate**		**Multivariate**	
	**Odds ratio**	**95% C.I**.	**Odds ratio**	**95% C.I**.
Age (advance by 1 year)	1.027	1.023–1.030	1.019	1.015–1.023
CCI >0	1.567	1.402–1.751	1.154	1.019–1.306
**Injury types**			
Head injuries (%)	1.155	1.019–1.309	0.934	0.793–1.101
Thoracic injuries (%)	0.7	0.613–0.800	0.811	0.7–0.939
Abdominal and retroperitoneal injuries (%)	0.328	0.265–0.406	0.475	0.376–0.601
Pelvic injuries (%)	0.58	0.442–0.762	0.8	0.599–1.069
Spinal cord injuries (%)	2.244	1.928–2.613	1.994	1.659–2.396
Extremity injuries (%)	0.684	0.601–0.779	0.803	0.698–0.923
**Index admission outcomes and complications**			
Stroke (%)	2.421	1.617–3.626	1.433	0.937–2.193
Acute coronary syndrome (%)	3.472	1.234–9.766	1.555	0.521–4.642
Prolonged mechanical ventilation (%)	3.073	2.706–3.489	1.313	1.121–1.538
Prolonged ICU LOS (%)	3.047	2.742–3.387	1.904	1.65–2.198
Prolonged LOS (%)	2.88	2.587–3.205	1.743	1.529–1.986

## Discussion

Long-term outcomes after trauma have long been an issue but have not been frequently discussed. A significant obstacle is that the follow-up after the index admission is not easily defined. Nonadherence after discharge from the index admission is common, especially for more severely injured patients who need outpatient services from multiple subspecialties (21). Hence, it would be challenging to design a study to examine the outcomes of these patients. The vastness of the NHIRD could compensate for this problem, but the shortcoming is that this database is low on details (22), so it might be insufficient to review certain outcomes at an individual level. However, in the scope of a nationwide study, the NHIRD could provide considerable information to investigate the panorama of the long-term results of major trauma.

We discovered several factors that might predict the need for LTC, including preexisting factors, injury types, and complications during index admission. None of these risk factors were modifiable, so therefore, we could not prevent major trauma patients from going into LTC. The value of these factors lies in the fact that if we could identify these risk factors early in the course of admission, it would be beneficial for communicating with the patients and their families. In Taiwan, the process of discharging patients with residual functional disabilities that require long-term care can be complicated. Often, the patients and their families could become reluctant to do so (23). Most hospitals in Taiwan suggest early recognition and communication to prepare the patients and their families for the forthcoming situation (24, 25). Recognizing the risk factors in our current study would facilitate the communication of our discharge plan.

Of the preexisting factors, age advancement and comorbidities were related to an increased likelihood of LTC. Our previous study (1) indicated that age was a prognostic factor associated with in-hospital mortality, whereas preinjury comorbidities were not related to short-term survival. These results might imply that Taiwan's healthcare system can salvage a patient's life with comorbidities from major trauma but cannot guarantee a recovery of quality of life. Similar results have been found in stroke patients in Taiwan, and the solution to this phenomenon was the post-acute care program provided by the NHI, which significantly improved the functional outcomes of stroke patients (26, 27). Lacking a bridge to post-discharge care among trauma patients is not a problem unique to Taiwan (28), and perhaps a more systemic approach to this issue might improve the quality of life of trauma patients.

Regarding injury types, spinal cord injuries were associated with an increased risk for LTC. The long-term quality of life for patients with spinal cord injuries is often unsatisfactory, as they have a high incidence of complications, including urinary incontinence, respiratory infections, pressure sores, and chronic pain (29, 30). Unsurprisingly, a significant portion of these patients require skilled LTC. In contrast, chest injuries, abdominal and retroperitoneal injuries, pelvic injuries, and extremity injuries were negatively correlated with the need for LTC. In our previous study (1), these injury types were also less likely to be associated with in-hospital mortality, indicating that these injuries were more treatable.

In terms of complications during the index admission, HAP, UTI, PMV, PICULOS, and PLOS were identified as independent risk factors for LTC in the future. HAP and UTI are both common hospital acquired infections are largely associated with frailty (31, 32). Frailty is often related to the use of LTC (33), and it is reasonable to assume that HAP and UTI were the presentation of frailty, which was the true factor that contributed to post-discharge LTC. Interestingly, PMV was related to LTC, whereas tracheostomy was not. A possible explanation of our data was that tracheostomy increased the probability of weaning from mechanical ventilation, providing a protective effect against PMV and making PMV an independent risk factor for LTC. However, this might not be the case in Taiwan. Note the disproportional distribution of tracheostomy and PMV: almost 13% of our study population experienced PMV, while only 0.5% received a tracheostomy. For cultural reasons, Taiwan's willingness to receive tracheostomy is low (34). Therefore, a more realistic explanation is that the few people who underwent tracheostomy might have masked its potential negative impact, thus leaving PMV the most decisive respiratory factor related to LTC.

PICULOS and PLOS were both related to LTC 1 year after discharge in the multivariate analysis. It is expected that PICULOS would lead to PLOS, but if both were identified as independent risk factors, then the underlying reasons PLOS resulted in LTC might differ from the reasons PICULOS did. Multiple medical or surgical factors have been related to PICULOS in trauma patients (35, 36). However, the cause of PLOS in trauma patients could be more complicated. Besides medical or surgical issues, socioeconomic factors, such as ethnicity or insurance status, could also lead to PLOS (37, 38). However, one should not conclude that these socioeconomic factors lead to PLOS during the index admission and eventually result in LTC in the future. It would be a stretch to make such an inference based on our current data, so further studies need to analyze the relationship between the long-term outcomes of trauma patients and their socioeconomic status.

The cost of LTC is another critical issue of our study, but we could not go into such a discussion without understanding the long-term survival of LTC and non-LTC groups. The median 10-year survival was inferior for the LTC group, yet the median 10-year health expenditure was still higher, indicating that the annual health expenditure was even higher for each individual in the LTC group. An interesting part of the survival analysis is that the survival probability for the LTC group was superior to the non-LTC group prior to post-discharge year seven, contrary to our shared belief that long-term survival should be superior for patients without the need for LTC. The exact reason for this result is somehow unknown and is beyond the context of this article, but some works of literature suggested that LTC might benefit survival in selected conditions (39, 40).

The cost of medical charges can vary dramatically between countries, so it may not be appropriate to compare the numbers directly. For example, Davis et al. reported that the mean cost of the 6-month post-discharge expenditure in all levels of trauma patients was 19,895 USD (3), while our data revealed that the average annual cost for post-discharge care for patients with ISS≥16 was merely 2,556 USD. A more similar comparison to Taiwan is Korea, which is also a nation with universal health insurance. Lim et al. reported that the annual cost for an injury episode in Korea, regardless of severity, was 3,075 USD in 2006 (41). However, this number included the expense of the index admission, which was excluded in our dataset. The differences in the designs of the two studies make it difficult to make a head-to-head comparison between Taiwan and Korea, but one can still see the noticeable differences between countries with universal health insurance and countries that rely on private health insurance.

In Taiwan, the development of LTC came pretty late. In the early 1990s', the debate was limited to preserving income rather than the care service. The government carried out some small LTC projects occasionally, but these projects were all experimental and did not last. It was not until 2007, when the government established the 10-Year Long-Term Care Project, commonly referred to as the LTC 1.0 by the public, that we had a large-scale LTC program. The LTC 1.0 plan required that local governments set up care management systems and develop various home or community services. However, the LTC 1.0 had two significant drawbacks: lacking funding and a shortage of caring facilities. Therefore, the actual application rate was quite low. After 10 years of debate, LTC 2.0 was finally launched, emphasizing expanding facilities and services to obtain easier access to LTC. Currently, both public and private facilities are involved in the LTC 2.0, and the adoption of smaller community-based facilities has enhanced the multifunctionality of LTC 2.0. Taiwan's LTC expenditure had been stationary in the LTC 1.0 era, but it started to rise dramatically since LTC 2.0 came into effect (42, 43). Our study period was long enough to cross the pre-LTC era, LTC 1.0 era, and LTC 2.0 era, witnessing the change over time.

We did not try to calculate the precise days of LTC for each patient because the daily functions of chronic patients could improve if given the right interventions (44), and they could also decline over time (45). The functional fluctuation on such patients is a chronic, dynamic, long-term process, likely necessitating both acute and chronic services (46). In this circumstance, it would be difficult to specify the duration and cost of LTC for these patients. Instead, we analyzed the total medical expenditure of our patients to obtain a more panoramic view. Compared to the internal data in Taiwan, the mean national annual healthcare expenditure on medical services between 2010 and 2020 was 7,316,66,667 USD per year, which translated to 318 USD per person (47). Our dataset showed that the average annual healthcare expenditure for each major trauma patient was 5,772 USD in the LTC group and 1,934 USD in the non-LTC group, which indicated that the healthcare expenditure was 18 times that of an average person for patients who needed LTC and six times for the non-LTC group. The mean annual healthcare expenditure of all major trauma patients in our cohort consumed 0.37% of the national annual healthcare expenditure on hospitals, clinics, and nursing facilities, while these people only accounted for 0.04% of the national population. We could use the cost of trauma care in the United States as an index to compare the proportion of healthcare spending used on trauma patients. Weir et al. published an article addressing the cost of trauma healthcare in the USA in 2005 (48). The total 1-year treatment cost of adult major trauma in the USA was estimated to be US$27 billion annually. Fifty-eight percent of these costs were accounted for by the index hospitalization, leaving 42% of the costs to be taken up by the annual post-discharge care, which is 11.34 billion USD.

Meanwhile, the US national healthcare expenditure on hospitals, clinics, and nursing facilities was 1,154.7 billion USD in 2005 (49). Therefore, we estimate that the post-discharge cost of major trauma patients in the US accounts for 0.98% of the healthcare expenditure. By comparing the two countries, we conclude that the post-discharge care for major trauma patients in Taiwan might seem to take up a significant portion of the healthcare budget but is still characterized by under-reimbursement. This issue must be addressed, and stakeholders should seek a solution to invest in post-discharge care for trauma patients for the benefit of individual patients and the nation.

There are several limitations to this study. The most significant shortcoming is that the NHIRD did not record trauma severity data, such as the Abbreviated Injury Score or the ISS. Therefore, we fell short of analyzing the potential effects of injury severity, and this drawback inhibited us from having a more in-depth discussion. However, from a public health point of view, it depicted a general understanding of LTC and the medical expense for major trauma patients. For patient selection, major trauma patients were extracted by the CIC. The application for CIC is sent by the physicians and the hospitals. In some cases, physicians might omit the application for CIC for their patients with major trauma. Therefore, the exact volume of major trauma patients could be underestimated by our patient selection method. However, using CIC as a proxy for major trauma is our only mean in the NHIRD because the ISS score is not documented in the NHIRD. In the economic burden analysis, the healthcare expenditure in the NHI was counted in point values, where 1 point should approximately equal 1 TWD. However, the payment of the NHI goes by a floating value system, and the actual amount of money reimbursement was mostly less than the point value demonstrated (50). However, the National Health Expenditure yearbook 2020 was counted in the actual amounts. Therefore, the proportion of the expenditure on post-discharge trauma care should be less than what we estimated. Another potential bias is that some long-term medical expenses were out-of-pocket payments by the patients or by private insurance. These payments were not covered by the NHI; hence, they were not presented in the NHIRD. Therefore, our calculation would somewhat underestimate the actual medical expense for long-term care after major trauma, also leading to an underestimation of the proportion of the expenditure on post-discharge trauma care. We also have to point out that 2003 to 2017 is a very long period, during which clinical practice might evolve, even to the point of altering the outcomes of trauma patients, eventually leading to differences in LTC expenditures. Taking splenic injury as an example, nonsurgical treatment gained popularity between 2002 and 2013 in Taiwan, rendering splenectomy a less popular option (51). Splenectomy is associated with an increased risk of pneumonia in the future (52), so less splenectomy might lead to less post-discharge healthcare expenses. The expenditure analysis based on the past might not represent the present situation. Lastly, the data in the NHI were payment-oriented, not quality-oriented; therefore, little data could be used to analyze the quality of life.

## Conclusions

16.14% of Taiwanese major trauma patients require LTC at least 1 year after being discharged. Independent risk factors for LTC include advanced age, comorbidities, spinal cord injury, PMV, PICULOS, and PLOS. The average annual healthcare expenditure per patient was 18 times that of an average person in Taiwan for the LTC group. The total economic burden for post-discharge LTC for major trauma patients was only 0.37% of the national annual healthcare expenditure. To achieve better care, the NHI should seek resolutions to invest in post-discharge care for major trauma patients.

## Data Availability

The data analyzed in this study is subject to the following licenses/restrictions: the datasets generated and/or analyzed during the current study are not publicly available due to the patient confidentiality restrictions by the NHIRD, but are available from the NHIRD on reasonable request. Requests to access these datasets should be directed to https://www.apre.mohw.gov.tw/.
