# Supplementary material for: Risk factors and economic impact of long-term nursing care after major trauma
Source: Front Public Health. 2025 Mar 18;13:1535784. doi: 10.3389/fpubh.2025.1535784 (PMC11958981; doi:10.3389/fpubh.2025.1535784)
Supplement: Supplementary file 1 [file Table_1.docx]

Supplement file: The International Classification of Diseases, Ninth Revision (ICD-9) codes use for the injury diagnosis of our study.

| Injury type | ICD-9 codes |
| --- | --- |
| Head injuries | 800-804; 850-854; 900.0; 900.8; 900.9 |
| Thoracic injuries | 807; 860-862; 805.2; 805.3; 901 |
| Abdominal and  retroperitoneal injuries | 863-866; 868; 869; 805.4; 805.5; 902.0-902.4 |
| Pelvic injuries | 867; 808; 805.6; 805.7; 902.5; 902.81; 902.82 |
| Spinal cord injuries | 952; 953; 806 |
| Extremity injuries | 812-813; 820-821; 823-824; 810-811; 818; 822; 827; 903; 904 |
| Thermal injuries | 940-949; 991 |
